# Closing the gaps on the viral photosystem‐I *psaDCAB* gene organization

**DOI:** 10.1111/1462-2920.13036

**Published:** 2015-10-14

**Authors:** Sheila Roitman, José Flores‐Uribe, Alon Philosof, Ben Knowles, Forest Rohwer, J. Cesar Ignacio‐Espinoza, Matthew B. Sullivan, Francisco M. Cornejo‐Castillo, Pablo Sánchez, Silvia G. Acinas, Chris L. Dupont, Oded Béjà

**Affiliations:** ^1^ Faculty of BiologyTechnion – Israel Institute of TechnologyHaifaIsrael; ^2^ Department of BiologySan Diego State UniversitySan DiegoCAUSA; ^3^ Department of Molecular and Cellular BiologyUniversity of ArizonaTucsonAZUSA; ^4^ Department of Ecology and Evolutionary BiologyUniversity of ArizonaTucsonAZUSA; ^5^ Departament of Marine Biology and OceanographyInstitute of Marine Sciences (ICM), CSICBarcelonaSpain; ^6^ Microbial and Environmental Genomics GroupJ Craig Venter InstituteSan DiegoCAUSA; ^7^ Present address: Department of MicrobiologyOhio State UniversityColumbusOH43210USA

## Abstract

Marine photosynthesis is largely driven by cyanobacteria, namely *Synechococcus* and *Prochlorococcus*. Genes encoding for photosystem (PS) I and II reaction centre proteins are found in cyanophages and are believed to increase their fitness. Two viral PSI gene arrangements are known, *psaJF→C→A→B→K→E→D* and *psaD→C→A→B*. The shared genes between these gene cassettes and their encoded proteins are distinguished by %G + C and protein sequence respectively. The data on the *psaD→C→A→B* gene organization were reported from only two partial gene cassettes coming from Global Ocean Sampling stations in the Pacific and Indian oceans. Now we have extended our search to 370 marine stations from six metagenomic projects. Genes corresponding to both PSI gene arrangements were detected in the Pacific, Indian and Atlantic oceans, confined to a strip along the equator (30°N and 30°S). In addition, we found that the predicted structure of the viral PsaA protein from the *psaD→C→A→B* organization contains a lumenal loop conserved in PsaA proteins from *Synechococcus*, but is completely absent in viral PsaA proteins from the *psaJF→C→A→B→K→E→D* gene organization and most *Prochlorococcus* strains. This may indicate a co‐evolutionary scenario where cyanophages containing either of these gene organizations infect cyanobacterial ecotypes biogeographically restricted to the 30°N and 30°S equatorial strip.

## Introduction

Cyanobacteria play a key role in oceanic photosynthesis and contribute to the global carbon cycle and oxygen supply (Li *et al*., 1993; Liu *et al*., 1997; Partensky *et al*., 1999). Genes encoding for photosystem‐II (PSII) reaction centres (the D1 and D2 proteins encoded by the *psbA* and *psbD* genes, respectively) are found in cultured and uncultured phages that infect marine cyanobacteria (Mann *et al*., 2003; Lindell *et al*., 2004; 2005; Millard *et al*., 2004; Sullivan *et al*., 2005; 2006; Zeidner *et al*., 2005; Sharon *et al*., 2007), are expressed upon infection (Lindell *et al*., 2005; 2007; Clokie *et al*., 2006), and it was suggested that this increases phage fitness (Bragg and Chisholm, 2008; Hellweger, 2009). See Puxty and colleagues (2014) for a recent review on viral ‘photosynthesis’.

Using environmental metagenomics, uncultured cyanophages were recently found to contain gene cassettes coding for photosystem‐I (PSI) reaction centres (Sharon *et al*., 2009). Two viral PSI gene organizations are currently known (Sharon *et al*., 2009; Béjà *et al*., 2012), *psaJF→C→A→B→K→E→D* and *psaD→C→A→B*. The *psaJF→C→A→B→K→E→D* cassette contains a gene fusion between the *psaJ* and *psaF* and is characterized by a low %G + C content of around 40%, while the four gene cassette, *psaD→C→A→B*, tends to have a higher %G + C content, ranging from 42% to over 50%. The fused PsaJF protein from the low %G + C cassette was hypothesized to be able to accept electrons not only from PSII (via plastocyanin or cytochrome c_6_) but to also work with other electron donors like soluble cytochrome c that usually function as electron donors to cytochrome oxidase (Sharon *et al*., 2009). This was recently shown using a heterologous *Synechocystis* system mimicking the cyanophage system (Mazor *et al*., 2014).

**Figure 1:**
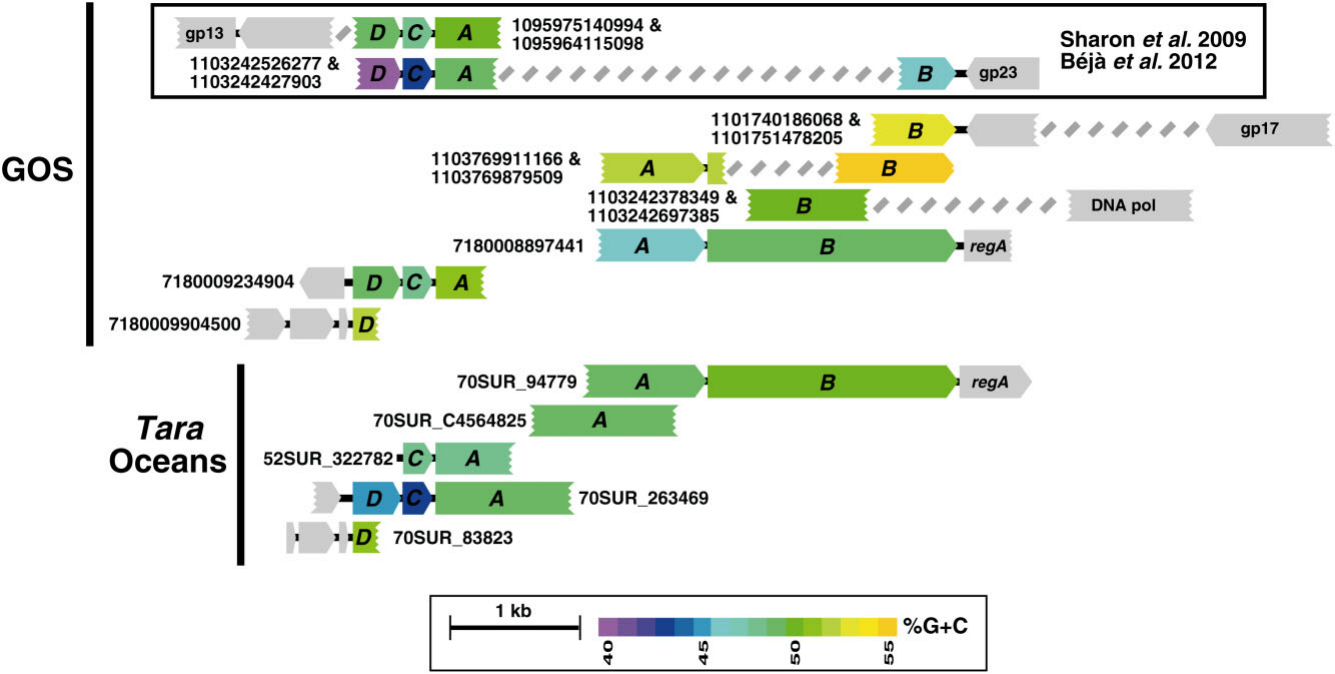
Schematic gene organization of GOS and *Tara* Oceans scaffolds containing viral PSI genes from the *psaD→C→A→B* cassette. PSI genes are coloured according to their %G + C content; the calculation was performed on each gene separately. Grey boxes represent viral ORFs. Two GOS clones previously reported are boxed at the top. DNA sequences can be found in Appendix S1. gp23 – major capsid protein; gp17 – terminase large subunit; DNApol – DNA polymerase; *regA* – translation regulator. For clarity, not all detected scaffolds are shown.

Our knowledge on the *psaD→C→A→B* gene cassette is based solely on two metagenomic scaffolds originating from the Global Ocean Sampling (GOS) expedition (Sharon *et al*., 2009; Béjà *et al*., 2012) (upper Fig. 1). These scaffolds contain a partial *psaD* gene, the highly conserved *psaC* gene, the beginning of *psaA*, and one clone also contains the far end of *psaB* gene. In addition, we have recently shown, using polymerase chain reaction (PCR) with primers amplifying the unique viral gene organization *psaC→psaA* (found in both gene cassettes), that *psaA* genes coming from the viral *psaD→C→A→B* cassette are diverse (Hevroni *et al*., 2015).

The goal of this study was to expand our knowledge regarding PSI genes carrying phages, with a special emphasis on phages carrying the *psaD→C→A→B* gene organization. We wanted to better understand why such two different viral PSI gene organizations exist, whether they are capable of different functions and who are the potential cyanobacterial hosts. For this 370 marine stations from six metagenomic projects were analysed for the presence of viral PSI genes. Numerous viral scaffolds were found from both PSI gene organizations, and the analysis of the scaffolds matching the *psaD→C→A→B* gene organization enabled us to close the gaps in missing parts of the gene sequences. In addition we were able to model the viral PsaA protein encoded by the *psaD→C→A→B* gene organization and to find substantial structural differences to its PsaA counterpart from the viral *psaJF→C→A→B→K→E→D* organization.

## Results and discussion

To date, numerous low %G + C viral PSI cassette sequences have been identified (Sharon *et al*., 2009; Alperovitch‐Lavy *et al*., 2011). In contrast, only two high %G + C viral PSI scaffolds are currently known (upper Fig. 1). To increase our understanding of the high %G + C viral PSI gene arrangement and fill up the sequence gaps in the *psaD→C→A→B* cassettes, we have examined the GOS (Rusch *et al*., 2007; Yooseph *et al*., 2007), Pacific Ocean Virome (POV) (Hurwitz and Sullivan, 2013), *Tara* Oceans (Karsenti *et al*., 2011; Brum *et al*., 2015; Sunagawa *et al*., 2015), C‐MORE:BULA (Hewson *et al*., 2009), Moore Virome Project (The Gordon and Betty Moore Foundation Marine Microbial Initiative genomes), and Hawaii and Line Islands metagenomic datasets using the viral protein sequences of PsaD, PsaA and PsaB as queries.

**Figure 2:**
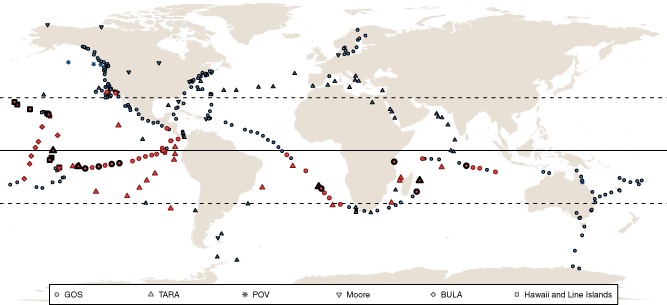
Map of stations analysed for the presence of viral PSI genes. Blue symbols indicate stations where viral PSI was not detected. Red symbols indicate stations where at least one viral PSI scaffold or read were found; bold red stations were positive for high %G + C viral PSI presence. Stations are indicated as circles for GOS; up triangles, *Tara* Oceans; down triangles, Moore Virome Project; diamonds, C‐MORE:BULA; asterisks, POV and squares, Hawaii and Line Islands. The equator is shown as a solid line, while latitudes 30°N and 30°S are shown as dashed lines.

Stations showing the presence of viral PSI genes (marked as red stations in Fig. 2) were mainly confined between 30°N and 30°S. It is important to remark that despite the differences between projects regarding sampling protocols, sequencing techniques, data analysis, etc., our results show that viral PSI gene cassettes are widespread. The presence of scaffolds matching the *psaD→C→A→B* cassette was observed in the Pacific and Indian oceans, and for the first time also detected in the Atlantic Ocean. To the same extent, it is worth noting the existence of different picocyanobacterial clades or ecotypes that occupy distinct environmental conditions (see Scanlan *et al*., 2009, for a review). For instance, within the *Synechococcus* subcluster 5.1 (Dufresne *et al*., 2008; Scanlan *et al*., 2009), to which most marine *Synechococcus* belong, just a few clades such as clade II and III (Scanlan *et al*., 2009) and clades CRD1 and CRD2 (Sohm *et al*., 2015) have been reported between 30°N and 30°S. On the contrary, clades I or IV are found in coastal and/or temperate mesotrophic open ocean waters largely above 30°N and below 30°S. The same geographical restrictions are valid for *Prochlorococcus* clades such as the HLII clade occupying strongly stratified surface waters between 30°N and 30°S, or the contrary case clade HLI living in more weakly stratified surface waters, particularly between 35° and 48°N and 35° and 40°S, just to mention a few examples (Scanlan *et al*., 2009). Therefore, it seems quite logical to assume that the distribution of cyanophages containing PSI‐viral genes would fit with the distribution of their cyanobacterial hosts, and in this particular case we suggest that cyanophages containing PSI‐viral genes are restricted to infect picocyanobacterial ecotypes living in the belt defined between 30°N and 30°S.

**Figure 3:**
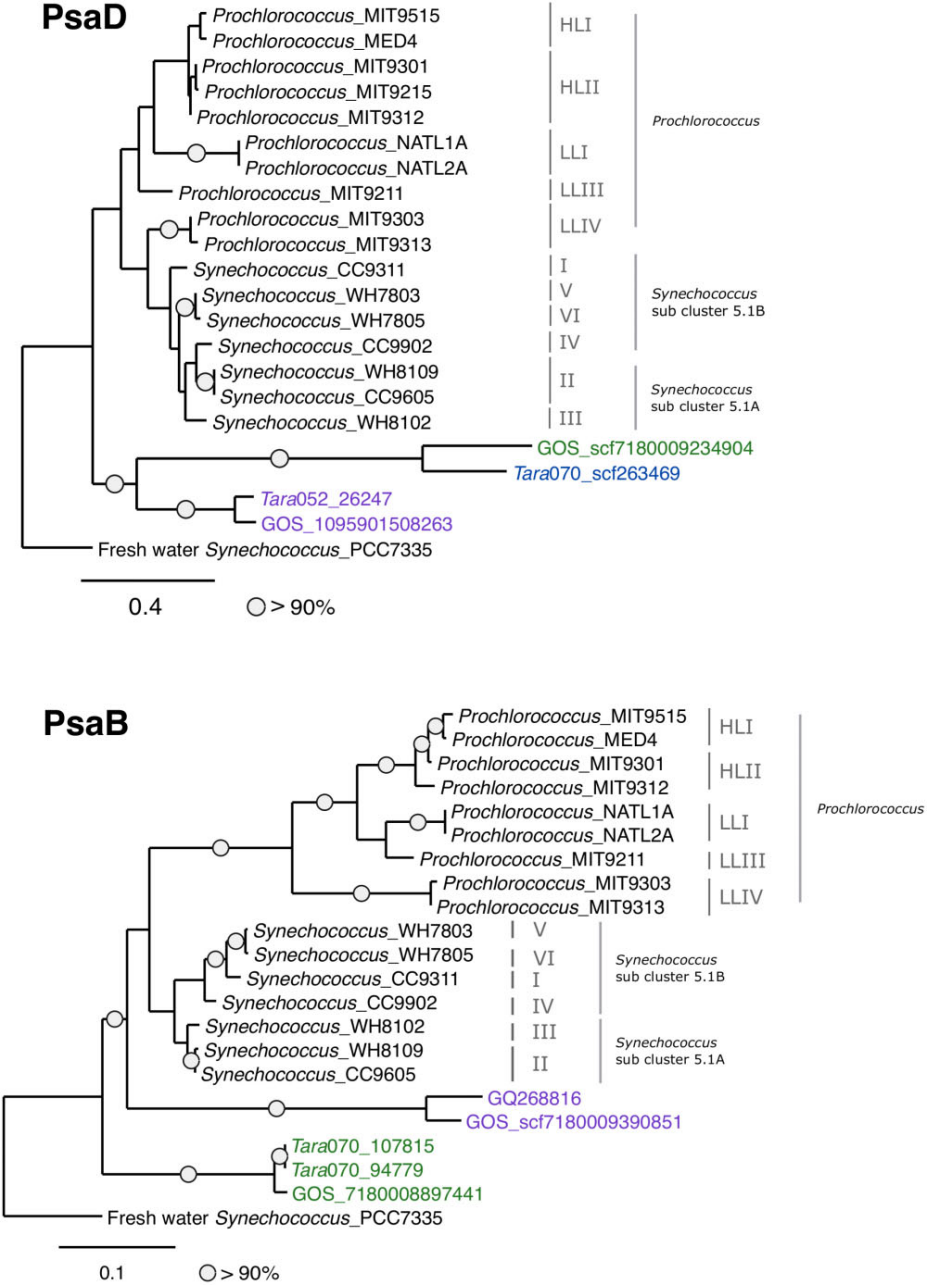
Maximum likelihood phylogenetic trees of (A) PsaD – based on 193 amino acids positions, and (B) PsaB – based on 756 amino acids positions. Circles represent bootstrap values higher than 90%. Phage name colours represent %G + C classification according to the colour index in Fig. 1, purple stands for low %G + C sequences, green and blue for high %G + C. The scale bar indicates the average number of amino acid substitutions per site.

The newly discovered scaffolds from the high %G + C gene organization allowed us, for the first time, to construct PsaA, PsaD and PsaB phylogenetic trees containing more than one viral high %G + C entity. As previously observed (Béjà *et al*., 2012), partial sequences of viral proteins from the high %G + C gene organization cluster together, within the marine *Synechococcus* clade, while partial proteins from the low %G + C gene organization group cluster separately forming their own clade. Having longer sequences originated from environmental scaffolds made it possible to construct full‐length PsaB and PsaD phylogenetic trees (Fig. 3), based on all 756 and 193 amino acids positions of PsaB and PsaD respectively. These trees show a different topology to the previously reported, with both low and high %G + C gene organizations forming monophyletic clades outside of the *Prochlorococcus* and *Synechococcus* clades.

Some of the new high %G + C scaffolds from GOS and Tara Oceans contained several viral genes other than PSI genes. These genes (e.g. DNA polymerase or *regA* genes) resemble genes from cyanomyophages (T4‐like phages; see Fig. S1), suggesting a possible *Myoviridae* origin for these scaffolds. In all viral scaffolds where sequences reaching beyond the photosynthetic gene arrangement borders were available, there were always viral genes constraining the arrangement (Fig. 1). This supports the notion that there are no other neighbouring photosynthetic genes accompanying the *psaD→C→A→B* gene arrangement. It is important to remark that the retrieved viral *psaD* sequences were always flanked by a non‐photosynthetic viral open reading frame (ORF), either upstream or downstream in the high %G + C and low %G + C gene organizations respectively. The same occurs downstream to the high %G + C *psaB* and upstream the *psaJF* (which can be found only in low %G + C sequences). Furthermore, the sequences retrieved consistently match the *psaJF→C→A→B→K→E→D* or *psaD→C→A→B* gene organizations, accordingly to their %G + C content, which might indicate that viral PSI genes are found solely in the two previously described cassettes. However, as metagenomic data are fragmented by nature, we cannot rule out the presence of standalone PSI photosynthetic genes (e.g. the *psaJ* gene; Sharon *et al*., 2011) or other yet‐unknown photosynthetic cassettes in the genomes of these cyanophages.

**Figure 4:**
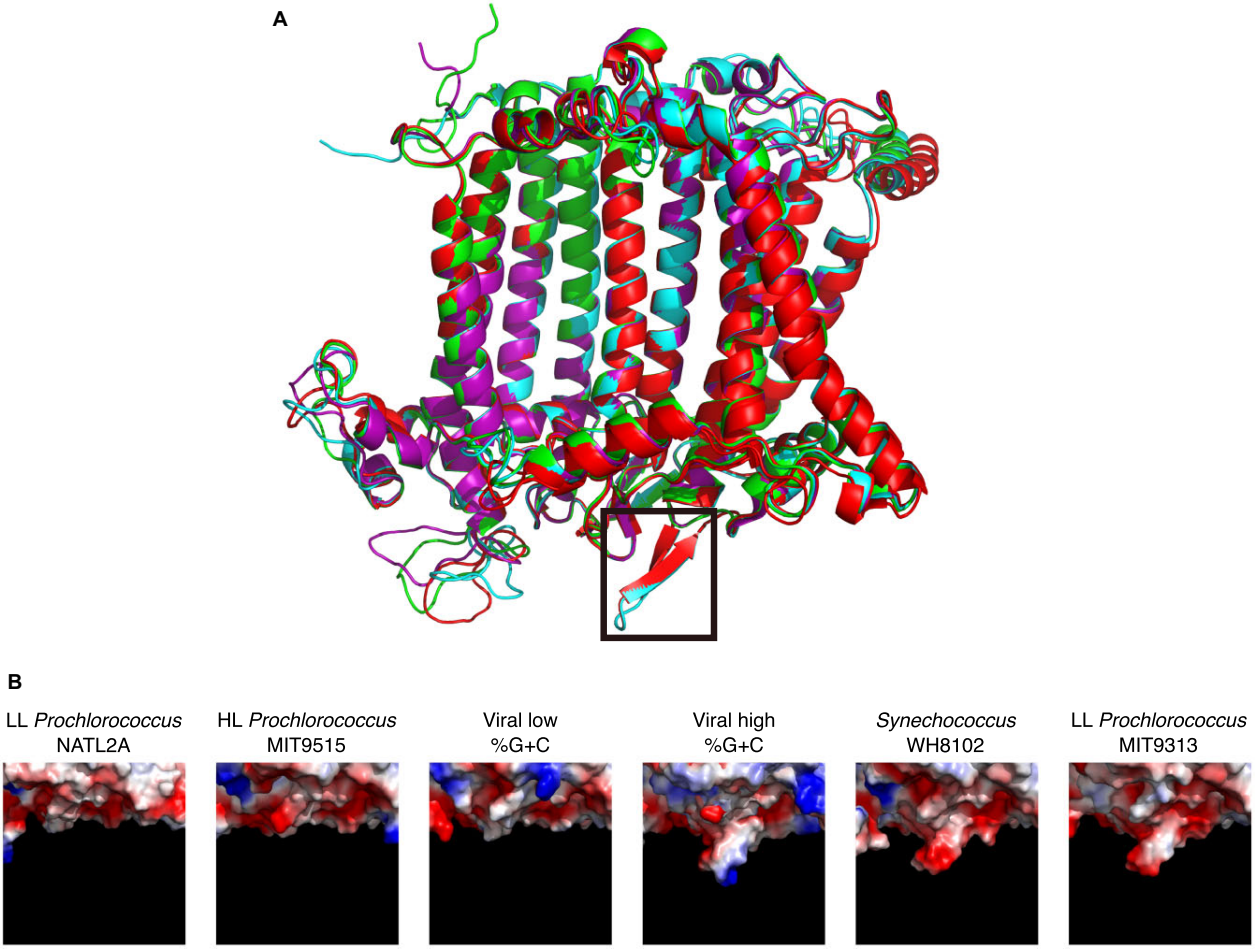
Structure modelling of PsaA proteins from cyanobacteria and cyanophages. (A) PsaA from *Synechococcus* (in cyan), HL *Prochlorococcus* (green), low %G + C viral (purple) and from the reconstructed high %G + C viral (red). The loop missing in PsaA from *Prochlorococcus* (except in LL *Prochlorococcus* MIT9313 and MIT9303) and the low %G + C viral, but present in *Synechococcus*, LL *Prochlorococcus* MIT9313 and MIT9303, and in the high %G + C viral PsaA is boxed. (B) Electrostatic potential of the lumenal side of PsaA proteins boxed in panel A. Red and blue indicate negative and positive potentials respectively. The loop sequences alignment can be found in Fig. S2.

Based on data from the environmental scaffolds, we were able to assemble a full‐length viral PsaA protein sequence from the high %G + C family by overlapping partial sequences. Structure prediction of the assembled viral PsaA was then compared with that of PsaA proteins from *Synechococcus*, *Prochlorococcus* and with the viral low %G + C. As shown in Fig. 4A, the overall structure of the four proteins is conserved except for a small loop (boxed in Fig. 4A) facing outside the membrane. This loop is in close proximity to the hydrophobic binding site of plastocyanin/cytochrome c_6_ (Sommer *et al*., 2004; Mazor *et al*., 2012), therefore potentially influencing the electron transfer between the electron donor and P700 in PSI. To further confirm the existence of this loop, we designed a set of degenerate primers based on a PsaA protein's sequence alignment and used them to amplify the gene from viral concentrates collected from the Line Islands. Positive overlapping *psaA* PCR products were successfully retrieved (GenBank #s KP411049‐KP411210), and their %G + C content was similar to viral low and high %G + C groups. This loop is found in the viral high %G +C PsaA, in *Synechococcus*, and also in low light adapted (LL) *Prochlorococcus* MIT9313 and MIT9303, and is missing in PsaA proteins of other LL *Prochlorococcus*, high light adapted (HL) *Prochlorococcus*, and in the viral low %G+C version. Interestingly, the high %G+C viral version of the loop is different from the marine cyanobacterial loop, containing a conserved arginine residue (Fig. S2); the viral loop is therefore positively charged compared with the cyanobacterial versions (Fig. 4B). Interestingly, viral encoded plastocyanin (PetE) has a lower isoelectric point (and thus potentially being negatively charged) compared with the host plastocyanin (Puxty *et al*., 2014). We suggest that the viral PetE version may have a higher affinity for the viral version of PsaA, possibly because of the lumenal loop of the phage's PsaA. It is important to note that no *petE* genes were found on any of the viral PSI metagenomic scaffolds retrieved in this study. Alternatively, the change in the viral PsaA loop may perhaps have a similar function as the fused PsaJF protein found in the viral *psaJF→C→A→B→K→E→D* cassette, namely being promiscuous for its electron donors and being able to accept electrons from electron donors other than plastocyanin or cytochrome c_6_ (Mazor *et al*., 2014).

We suspect that cyanophages carrying high %G + C PSI genes infect hosts with similar protein structures, leading to the assumption that phages carrying the *psaD→C→A→B* gene organization might infect cyanobacteria which have the lumenal loop in PsaA and have a similar geographical distribution. The hosts could potentially be *Prochlorococcus* MIT9313 and MIT9303 [belonging to an LL *Prochlorococcus* clade (clade LLIV)], which is considered to be closely related to *Synechococcus* and is a clade widely distributed within the 40°N to 35°S latitudinal range, largely restricted to the deep euphotic zone (Scanlan *et al*., 2009; Biller *et al*., 2015), or *Synechococcus* from clades CRD1 and CRD2 which are present in similar latitudes, 40°N to 30°S (Sohm *et al*., 2015).

The lumenal loop is missing in PsaA protein versions originating from the viral *psaJF→C→A→B→K→E→D* cassette. This could indicate that cyanophages containing the *psaJF→C→A→B→K→E→D* gene organization might infect different cyanobacterial hosts as compared with phages containing the *psaD→C→A→B* gene organization. Likewise, these cyanophages would only infect cyanobacterial species or ecotypes latitudinally restricted to the strip defined between 30°N and 30°S. However, we cannot rule out the possibility that these two kinds of phages infect the same hosts but perform differently to achieve a similar outcome, namely PsaJF and the lumenal loop in PsaA could perform similar functions regarding the docking of electron donors to PSI.

Our PsaA modelling suggests that the PsaA protein from the *psaD→C→A→B* gene organization might function differently from PsaA versions of the potential hosts and the other viral gene organization, presenting a new kind of PSI complex. Evolutionary studies regarding PSI proposed that a minimal complex composed by the PsaA, PsaB, PsaC and PsaD proteins could theoretically form a functional reaction centre (Nelson, 2011). Therefore, characterizing phages with the *psaD→C→A→B* gene set might shed light on PSI evolution and lead to a better understanding of PSI light reactions, as this might be the only extant case of a minimal, functional PSI that comprised only four subunits.

## Experimental procedures

### Metagenomic data analysis

Microbial and viral metagenomic datasets were downloaded from CAMERA (Seshadri *et al*., 2007), iMicrobe database (http://imicrobe.us) or MG‐RAST (Meyer *et al*., 2008).

Microbial metagenomes from the GOS expedition project (Venter *et al*., 2004; Rusch *et al*., 2007), Hawaii and Line Islands, Biogeochemistry of the Upper Ocean: Latitudinal Assessment (C‐MORE:BULA) project (Hewson *et al*., 2009), *Tara* Oceans expedition (Sunagawa *et al*., 2015), and viral metagenomes from the POV project (Hurwitz and Sullivan, 2013), Moore Virome project, and *Tara* Oceans expedition virome (Brum *et al*., 2015) were analysed using blast v2.2.28^+^ tools (see Table S1 for the metagenomic datasets).

First a collection of amino acid sequences from low and high %G + C viral PSI genes *psaA* and *psaB* (Table S2) was used as query for a tblastn search (e‐value 0.1) against the metagenomes. Contigs and reads matching PSI proteins and their paired‐end mates were further screened using blastx (e‐value 10e‐10) against the NCBI non‐redundant (nr) protein database to identify those that were likely to have a viral origin according to the top score hit of taxonomy assignment and presence of viral genes on the contig or read mate.

### psaA amplification and cloning

Degenerate primers were designed against a PsaA protein multiple sequence alignment of a wide variety of organisms, including eukaryotes, prokaryotes and viral PsaA proteins obtained from GenBank (Primers TTTW[I/V]W_fwd, ACNACNACNTGGRTNTGGAA; HHIHAF_rev, RAANGCRTGDATRTGRTG; MPPY[P/A]Y_fwd, ATGCCNCCNTAYSCNTA; TTW[A/S]FF_rev, RAARAANBWCCANGTNGT; with 512, 192, 256 and 1536 degeneracy respectively). Two PCR reactions (Reaction B: TTTW[I/V]W_fwd – HHIHAF_rev; Reaction L: MPPY[P/A]Y_fwd – TTW[A/S]FF_rev) were performed directly on viral concentrates from the Pacific Southern Line Islands [collected in April 2009 and in November 2013 from Caroline island (Millennium Island) and in October 2013 from Vostok Island]. Viral concentrates were prepared according to Haas and colleagues (2014). The PCR reactions B and L were performed using BIO‐X‐ACT^TM^ Short mix (Bioline, London, UK), in a total volume of 30 μl containing 1 μl of phage concentrate as template, OptiBuffer (1×), 2 μM primers (each), 0.8 mM dNTPs, 2 mM MgCl_2_ and 2.4 U BIO‐X‐ACT^TM^ Short DNA polymerase. The PCR conditions were the following: Reaction B – 95°C, 5 min; 40 cycles of 95°C, 30 s; 53°C, 30 s, 72°C, 100 s; and Reaction L – 95°C, 5 min; 40 cycles of 95°C, 30 s; 50°C, 30 s, 72°C, 70 s. Reaction L PCR was also performed using the Tiangen 2× Taq PCR MasterMix (Tiangen Biotech, Beijing), in a total volume of 25 μl containing 1 μl phage concentrate as template, 1.2 μM primers (each) and Master Mix (1×). The PCR amplification conditions were as previously described. The PCR products (Reaction B – 1500 bp approximately; Reaction L – 1100 bp approximately) were cloned using the PCRII‐TOPO vector (Invitrogen, San Diego, CA) according to the manufacturer's specifications and sequenced using Sanger sequencing (Macrogen Europe, Amsterdam, NL). Sequences retrieved were checked against published *psaC→A* viral sequences using k‐mers analysis in the overlapping region between the amplicons (Hevroni *et al*., 2015).

### Phylogenetic tree construction and analysis

PsaB and PsaD sequences from the *Tara* Oceans and GOS projects were obtained by translating the scaffold DNA sequence according to the correct open reading frame and aligned along with sequences from *Prochlorococcus* and *Synechococcus* (retrieved from GenBank). Multiple sequence alignments were constructed using ClustalX v2.1 (Larkin *et al*., 2007). Maximum likelihood phylogenetic trees were constructed using the phylogeny.fr pipeline (Dereeper *et al*., 2008), which included PhyML v3.0 (Guindon *et al*., 2010) and the WAG substitution model for amino acids (Whelan and Goldman, 2001). One hundred bootstrap replicates were conducted for each analysis. See Appendices S2–S7 for the alignments used to construct the trees.

### PsaA protein structure models

Structural models for the viral, *Prochlorococcus* and *Synechococcus* PsaA proteins were predicted and folded according to the Protein Data Bank 1JB0 record (Jordan *et al*., 2001) using the HHPred software v2.0.16 (Soding *et al*., 2005) and Modeller v9.11 (Sali *et al*., 1995). Protein models were visualized and the electrostatic potential calculated using PyMOL (The PyMOL Molecular Graphics System, Version 1.5.0.4 Schrödinger, LLC.). For each protein model, we performed several protein predictions and selected one representative sequence (in bold) for Fig. 4 (*Synechococcus* WH7803, **WH8102**, WH7805, WH8109, CC9902, RS9917, RS9916, CC9311, RCC307; HL‐*Prochlorococcus* CCMP1986, **MIT9515**, MIT9301; LL‐*Prochlorococcus* NATL2A, MIT9313, MIT9211; low %GC viral **GQ268816**; high %GC viral *psaA* sequences from Fig. 1 and five different sequences of PCR reactions B and L, among them **KP411157.1** and **KP411207.1**).

## Supporting information


**Fig. S1.** Maximum likelihood phylogenetic trees of (A) DNApol, (B) gp17, (C) gp23 and (D) RegA. Circles represent bootstrap values higher than 90%. Phage sequences retrieved in this study are coloured in red. The scale bar indicates the average number of amino acid substitutions per site.

**Fig. S2.** Multiple sequence alignment of the loop area in partial PsaA proteins. The arginine conserved in high %G + C viral sequences is marked in blue. Conserved negative amino acids are coloured in red. Names of the viral sequences represent reads/scaffolds or PCR products retrieved in this study (except for GQ268816).

**Table S1.** Metagenomic datasets analysed.

**Table S2.** Sequences used as query for the tblastn analysis.

**Appendix S1.** DNA sequences from Fig. 1.

**Appendix S2.** Protein alignment used to construct the PsaD phylogenetic tree (Fig. 3A).

**Appendix S3.** Protein alignment used to construct the PsaB phylogenetic tree (Fig. 3B).

**Appendix S4.** Protein alignment used to construct the DNApol phylogenetic tree (Fig. S1A).

**Appendix S5.** Protein alignment used to construct the gp17 phylogenetic tree (Fig. S1B).

**Appendix S6.** Protein alignment used to construct the gp23 phylogenetic tree (Fig. S1C).

**Appendix S7.** Protein alignment used to construct the RegA phylogenetic tree (Fig. S1D).
