# Supplementary material for: Closing the gaps on the viral photosystem‐I psaDCAB gene organization
Source: Environ Microbiol. 2015 Oct 14;17(12):5100–8. doi: 10.1111/1462-2920.13036 (PMC5019241; doi:10.1111/1462-2920.13036)
Supplement: Supplementary file 3 — Table S1. Metagenomic datasets analysed. [file EMI-17-5100-s003.docx]

| **Metagenome data set** | **Source** | **Project code/ Accession number** |
| --- | --- | --- |
| Global Ocean Sampling (GOS) | CAMERA (also available on iMicrobe) | CAM_PROJ_GOS |
|  | European Nucleotide Archive | ERX913362-ERX913706 |
| GOS Baltic Sea | iMicrobe | CAM_P_0001109 |
| GOS Banyoles | iMicrobe | CAM_P_0001174 |
| “Biogeochemistry of the Upper Ocean: Latitudinal Assessment” cruise (C-MORE: BULA) | iMicrobe | CAM_PROJ_Bacterioplankton |
| Moore Virome project | iMicrobe | CAM_PROJ_BroadPhage |
| Pacific Ocean Virome project (POV) | iMicrobe | CAM_P_0000915 |
| TARA Oceans microbial metagenome | European Nucleotide Archive | PRJEB1787 |
| TARA Oceans viral metagenome | European Nucleotide Archive | PRJEB1788 |
| Hawaii & Line Islands | MG - RAST | 4572816.3 |
|  |  | 4574475.3 |
|  |  | 4572817.3 |
|  |  | 4574476.3 |
|  |  | 4572818.3 |
|  |  | 4574477.3 |
|  |  | 4572819.3 |
|  |  | 4574478.3 |
|  |  | 4572820.3 |
|  |  | 4573625.3 |
|  |  | 4572821.3 |
|  |  | 4572822.3 |
|  |  | 4572823.3 |
|  |  | 4572824.3 |
|  |  | 4574479.3 |
|  |  | 4574480.3 |
|  |  | 4572825.3 |
|  |  | 4574481.3 |
|  |  | 4572826.3 |
|  |  | 4572827.3 |

**Table S1**. Metagenomic data sets analyzed.
