# Supplementary material for: Closing the gaps on the viral photosystem‐I psaDCAB gene organization
Source: Environ Microbiol. 2015 Oct 14;17(12):5100–8. doi: 10.1111/1462-2920.13036 (PMC5019241; doi:10.1111/1462-2920.13036)
Supplement: Supplementary file 4 — Table S2. Sequences used as query for the tblastn analysis. [file EMI-17-5100-s004.docx]

| **Sequence name** | **Aminoacid sequence** | **Reference** |
| --- | --- | --- |
| PsaA_JCVI_READ_1095964115098 | MTMTPEKKERSSQVEKVNVPATTELWRKPGWFDKSLKKGPETTTWIWNLHANAHDFDAHT  NDLQEVSRKIFAAHFGHLGVIFLWMSGFFFHSARFSNYSGWLADPTHIIPSSYK | (Sharon et al. 2009) |
| PsaA_JCVI_READ_1103242526277 | VEKVNIPATTELWRKPGWFSKTLKKGPETTTWIWNLHADAHDFDAHTNDLQEVSRKIFSA  HFGHLAVIFIWMSGFFFHSAKFSNYTCWLTDP | (Béjà et al. 2012) |
| PsaA_UniProt=B7T4F0 | MTITPPKKNPEDFVDKNPVPVSTEKWGKFGFYDKTLAKGPKTTTWIWNLHADAHDFDKHS  SDLEDNSRKIFAAHFAHLGIIFVWMSSFFFQGARFSNYTGWLADPTHVKPGGQVVWPILG  QEILNGDQGAGYHGLRITSGLFQMWGAWGITSEIELFALAFGALFMAALMFNAGAFHFHV  AAPKLSWFQNVNSMMNHHLAGLLGLGSLGWAGHLIHISIPTNTLLDAIDAGTPMVLNGRL  IETLTDIPPPHVLCSPEIASQIVPGLGSGVSNFFSLNWMAFSDLLTFKGGLNPVTGSLWM  TDIAHHHLAIAVMFIIAGHMYRTNWGIGQNLKDILDGQDGFPRGVTHRGLYEFLAESRHA  QLSLNLAMLGSISIIVSHHMYAMPPYPYLGIEYPTVVGLFTHHMWIGGFLIVGAGAHGSI  ALIRDYIPANHIGNVLDRIIKSRDAIISHLNWVCIFLGFHSFGLYIHNDTMRALGRPNDL  FSDTAIQLQPVFAQSIQRLHASLYNVSDVFGGTTTMVGGKIANAPFTLGTADFMIHHIHA  FQIHVVALILIKGVLYSRNSQLIPDKGKLGFRFPCDGPGRGGTCQVSGWDHVFLGLFWMY  NCISIVIFHFSWKMQDVWGLSGGNFAQSSITINGWLRDFLWSQSSQVLNSYGQDISMYGL  MFLGAHFVWAFSLMFLWSGRGYWQELIESIAWAHNKLKLAPTIQPRAMSITQGRAVGVAH  FLLGGIATTWAFFFAHLIPIS | Uniprot |
| PsaA_UniProt=B7T4D7 | HDFDKHSSDLEDNSRKIFAAHFAHLGIIFVWMSSFFFQGARFSNYTGWLADPTHVKPGGQ  VVWPILGQEILNGDQGAGYHGLRITSGLFQMWRAWGITSEIELFALAFGALFMAALMFNA  GAFHFHVAAPKLSWFQNVNSMMNHHLAGLLGLGSLGWAGHLIHISIPTNTLLDAIDAGTP  MVLNGRLIETLTDIPPPHVLCSPEIASQIIPGLGSGVSNFFSLNWMAFSDFLTFKGGLNP  VTGSLWMTDIAHHHLAIAVMFIVAGHMYRTNWGIGQNLKGILDGQDGFPQGVTHRGLYEF  LAESRHAQLSLNLAMLGSISIIVSHHMYAMPPYPYLGIEYPTVVGLFTHHMWIGGFLIVG  AGAHGSIALIRDYIPANHIGNVLDRILKSRDAIISHLNWVCIFLGFHSFGLYIHNDTMRA  LGRPNDLFSDTAIQLQPVFAQSIQRLHASLYNVSDVFGGTTTMVGGKIANAPFTLGTADF  MIHHIHAFQIHVVALILIKGVLYSRNSQLIPDKGKLGFRFPCDGPGRGGTCQVSGWDHVF  LGLFWMYNCISIVIFHFSWKMQDVWGLSGGNFAQSSITINGWLRDFLWSQSSQVLNSYGQ  DISMYGLMFLGAHFVWAFSLMFLWSGRGYWQELIESIAWAHNKLKLAPTIQPRAMSITQG  RAVGVAHKGEFHS | Uniprot |
| PsaA_UniProt=C7EDU5 | MTLTPPQKPQEDFVDKNPVPVSAEKWSKFGFFDKTLAKGPKTTTWIWNLHADAHDFDKHS  NDLQNNSRKIFAAHFAHLGIIFVWMSSFFYQGAKFSNYTGWLADPTHVKPGGQIVWPILG  QEILNGDQGAGYHGLRITSGLFQMWRGWGITSEIELYALAFGALFMAALMFNAGAYHFHV  SAPKLAWFQNVESMMNHHLAGLLGLGSLGWAGHLIHISIPTNTLLDAIDAGTPMVLNGRL  IETLTDIPPPHVLCSPSVASQIIPGLGSGVSNFFSLNWLAFSDFLTFKGGLNPVTGSLWM  TDIAHHHLAIAVMFIIAGHMYRTNWGIGHTLKEILDGQNGFPEGVTHRGLFEFLAESRHA  QLSLNLAMLGSISIIVAQHMYSMPPYPYLGIEYPTVVGLFTHHMWIGGFLIVGAGAHASI  AMIRDYNPVQHMGNVLDRLLKSRDAIISHLNWVCIFLGFHSFGLYIHNDTMRALGRPNDL  FSDTAIQLQPVFAQSVQRLHAALYNVSDVFGGTTTMVGGKIANAPFTLGTADFMIHHIHA  FQIHVVALILIKGVLYSRNSQLIPDKGKLGFRFPCDGPGRGGTCQVSGWDHVFLGLFWMY  NCISIVIFHFSWRMQDVWGLSGGNFAQSSITINGWLRDFLWSQSSQVLNSYGQDISMYGL  MFLGAHFVWAFSLMFLWSGRGYWQELIESIAWAHNKLKLAPTIQPRAMSITQGRAVGVAH  FLLGGIATTWAFFFAHLIPIS | Uniprot |
| PsaA_UniProt=U5LNQ9 | MTQAPEKRENQFVEKVNVPATTELWRKPGWFDKSLKKGPETTTWIWNLHANAHDFDAHTN  DLQEVSRKIFSAHFGHLGVIFLWMSGFFYHSARFSNYSGWLADPTHIKPSALQVWDVFGQ  GSLNGDMGGGFMGQQITSGLLHMYRAWGITSETQLLSLAVGALVMCGLMVNAGVFHYHVA  APKLEWFQNVESMLNHHLAGLLGLGSLSWAGHLIHVSVPMTKLM | Uniprot |
| PsaA_UniProt=U5LKD4 | MTLTPPQKPQEDFVDKNPVPVSAEKWSRFGFFDKTLAKGPKTTTWIWNLHADAHDFDKHS  NDLQNNSRKIFAAHFAHLGIIFVWMSSFFYQGAKFSNYTGWLADPTHVKPGGQIVWPILG  QEILNGDQGAGYHGLRITSGLFQMWRGWGITSEIELYALAFGALFMAALMFNAGAYHFHV  SAPKLAWFQNVESMMNHHLAGLLGLGSLGWAGHLTHISIPTNTLL | Uniprot |
| PsaA_UniProt=U5LLA7 | MTVTPPKKPQSELVDQNPVPVSTEKWGKPGFFDKSLSKGPKTTTWIWNLHADAHDFDKHS  SNQQENSRKIFAAHFAHLGIIFVWMSSFFFQGARFSNYTGWLADPTHVKPGAQVVWPILG  QEILNGDQGAGYHGIRITSGIFQMWRAWGITNETELMALALGALVMAAVMFNAGAYHFHI  AAPKLAWFQNVESMMNHHLAGLLGLGSLEWARHLIHISISTNMLM | Uniprot |
| PsaA_GQ268816 | VDKNPVPVSAEKWSKFGFFDKTLAKGPKTTTWIWNLHADAHDFDKHSNDLQNNSRKIFAAH  FAHLGIIFVWMSSFFYQGAKFSNYTGWLADP | (Sharon et al. 2009) |
| PsaB_JCVI_READ_1103242427903 | GWVTFYWHWNHLAIWSGNVAQFNESSTYVMGWFRDYLWLNSSQLINGYNPFGSNNLAVWS  WMFLFGHLVWATGFMFFISWRGYWQELIETIVWAHQRTPLANLAVFKDKPVALSIVQARV  VGLGHFTVGYILTYAAFLIAFTSGKFG | (Béjà et al. 2012) |
| PsaB_JCVI_READ_1101751478205 | TTTLVLVKGALDARGSKLMPDKKDFGYSFPCDGPGRGGTCDISAWDAFYLAVFWALNTIG  WVTFYWHWKHLAIWSGNVAQFNESSTYLMGWFRDYLWLNSSQLINGYNPFTSNNLAVWAW  MFLFGHLVWATGFMFLISWRGYWQELIETIVWAHQRTPLARMAVFKDKPVALSIVQARVV  GLGHFTVGYVLTYAAFLIASTSSKFG | (Béjà et al. 2012) |
| PsaB_UniProt=B7T4E0 | VAWQGNFEQFVADPQHVVPIAHRIIDPHFGQGATEAYTQAGATFTVNRLYSGIYHWWYTI  GLRTNVQLYAGGAFMAFLSLVSLSAANLHLQPKFRPTLAWFKDNENRLNHHLSVLFGFSS  IAWAGHLIHVAIPISRGIDINWSNYILTPPHPAGLLPFFTGNWAAYAQNPDGLNQVFGTT  EGSGTAILTFMGGFHPQTEALWLTDIAHHHLAIGAIFIIAGHMYRSMWGIGHSMKEILEA  HTPPRGGLGAGHKGLYETVTDSLHLQLGLALASLGVVTSLVAQHMYSMPSYAFISRAYTT  QATLWVHHQYIATALMVGAFAHGAIFFVRDYDPTLNKDNVLGRMLEHKEAIISHLSWVSL  FLGFHTLGIYVHNDVVMAFGHPERQILIEPIFAQWIQAASGKMMYGLSFLLSDPNSAASL  AAESMPGNHYWMSAMNDQTNSLFLPIGPADLLVHHAIALGLHTTTLILVKGALDARGSKL  IPDKKDLGYSFPCDGPGRGGTCDSSGWDAFYLSVFWAFNTIAWADFYWHWKNLASWQGNQ  AQFNESGTYLMGWFRDYLWANSAPVITGYTPFGMNNLSVWAWMFLFGHLVWATGFMFLIS  WRGYWQEWIETLVWAHNRTPLANIAGWRDKPV | Uniprot |
| PsaB_UniProt=B7T4D9 | MATKTKFPKFNQDLASDPTTRRLWYGLATAHDFESHDNMTEEKLYQKLFATHSGHLAIIF  LWVAGNLIHIAWQGNFEQFVADPQHVIPIAHRIWDPHFGQGATDAYTQAGSTFPVNRLYS  GLYHWWYTVGMRTNMQLYTGGGFMILLAMISILGAQLHLKPKFAPKLAWFKDNENRLNHH  LSVLFGFSSIAWAGHLIHVAIPASRGITVDWTNYVFMKPHPAGLLPFFTGNWGVYAQNPD  GINQIFNTTEGSGTAILTFMGGFHPQTEALWLTDIAHHHLAIGVIFIIAGHMYRSAWGIG  HSMKEILEAHTPPRGGLGVGHKGLYETVTDSLHLQLGLALASLGVVTSLVAQHMYSMPSY  AFIAQAHTTQAALYVHHQYIATALMVGAFAHGAIFFVRDFDPTLNKDNVLDRMLQHKEAI  ISHLSWVSLFLGFHTLGIYVHNDVVMAFGHPGRQILIEPIFAQWIQAASGKMMYGLSFLL  SDPNSAASLAADSMPGDHYWMSAINDQTNSLFLPIGPADLLVHHAIALGLHTTTLILVKG  ALDARGSKLIPDKKDLGYSFPCDGPGRGGTCDSSGWDAFYLSVFWAFNTIAWADFYWHWK  NLASWQGNQAQFNTSGTYLMGWFRDYLWANSAPVITGYTPFGMNNLSVWAWMFLFGHLVW  ATGFMFLISWRGYWQELIETLAWAHQRTPIANIAGWRDKPV | Uniprot |
| PsaB_UniProt=C7EDU6 | MATQTKFPKFNQDLASDPTTRRLWYGLATAHDFESHDNMNEEKLYQKLFSTHFGHLAIIF  LWVAGNLIHVAWQGNFEQFVADPQNVIPIAHRIWDPHFGQGATEAYTQAGSTFPVNRLYS  GLYHWWYTIGLRTNMQLYTGGGFMILLSMISILGAQLHLKPKFAPKLAWLKDNENRLTHH  LSVLFGFSSIAWAGHLIHVAIPASRGITVDWTNYVFMKPHPAGLLPFFTGNWGVYAQNPD  GLNQIFNTTEGSGTAILTFLGGFHPQTEALWLTDIAHHHLAIGVIFIIAGHMYRSYWGIG  HSMKEILEAHTPPRGGLGAGHKGLYETITDSLHLQLGLALASLGVVTSLVAQHMYSMPSY  AFISRAYTTQATLWVHHQYIATALMVGAFAHGAIFFVRDYDPTLNKDNVLGRMLEHKEAI  ISHLSWVSLFLGFHTLGVYVHNDVVMAFGHPERQILIEPIFAQWIQAASGKMMYGLSFLL  SDPNSAASLAADSMPGDHFWMNAINDKSNSLFLPIGSADLLVHHAIALGLHTTTLILVKG  ALDARGSKLIPDKKDLGYSFPCDGPGRGGTCDSSGWDAFYLSVFWAFNTIAWADFYWHWK  NLASWQGNQAQFNESGTYLMGWFRDYLWANSAPVITGYTPFGMNNLSVWAWMFLFGHLVW  ATGFMFLISWRGYWQELIETLVWAHQRTPIANIAGWRDKPVALSIVQARLVGLSHFVIGF  VLTYAPFLVATTASRYG | Uniprot |
| PsaB_GQ268816 | TGFMFLISWRGYWQELIETLVWAHQRTPIANIAGWRDKPVALSIVQARLVGLSHFVIGFVLTYAPFLVATTASRYG | (Sharon et al. 2009) |

**Table S2.** Sequences used as query for the TBLASTN analysis.
